# Efficacy of salmeterol and magnesium isoglycyrrhizinate combination treatment in rats with chronic obstructive pulmonary disease

**DOI:** 10.1038/s41598-022-16775-2

**Published:** 2022-07-19

**Authors:** Ye Yang, Lei Huang, Chongchong Tian, Bingjun Qian

**Affiliations:** Department of Pharmacology and Medicinal Chemistry, Jiangsu Vocational College of Medicine, Yancheng, 224005 Jiangsu People’s Republic of China

**Keywords:** Medical research, Pharmacology

## Abstract

The most classic treatment recommended in the current chronic obstructive pulmonary disease (COPD) guidelines is glucocorticoid and β2 receptor agonist combination, such as salmeterol xinafoate and fluticasone propionate (Sal/Flu), causing many adverse reactions due to hormones. Magnesium isoglycyrrhizinate (MgIG) is an anti-inflammatory glycyrrhizic acid preparation for treating chronic inflammation, contributing to its structure is similar to steroidal anti-inflammatory drugs. In this study, we successfully established COPD rat model by endotracheal-atomized lipopolysaccharide exposure and cigarette smoke induction, as characterized by lung function decline. We discovered that salmeterol xinafoate/MgIG combination could alleviated lung inflammation infiltration, airway wall thickness (AWT) and the secretion of bronchial mucin MUC5AC of COPD rats more than salmeterol xinafoate, MgIG, or salmeterol xinafoate and fluticasone propionate treatment did, as well as reduced inflammatory cells (white blood cells, neutrophils and lymphocytes) accumulation in bronchoalveolar lavage fluid and decreased TNF-α, IL-6 and IL-1β production in the serum of COPD rats. Finally, we found that Moreover, the mechanism involved might be related to the suppression of JAK/STAT signaling pathway. Overall, our studies suggested that MgIG might be a potential alternative adjuvant drug for fluticasone propionate for the clinical treatment of patients with COPD.

## Introduction

Chronic obstructive pulmonary disease (COPD), also known as obstructive emphysema, can further develop into common chronic diseases of pulmonary heart disease and respiratory failure^1,2^. Recently, the incidence rate of COPD has increased significantly due to air pollution, smoking and chronic bronchitis^3^. The symptoms of COPD cause a huge global burden of respiratory diseases^4^. COPD causes impaired activity, increased risk of hospitalization and worse symptoms including chronic bronchitis and emphysema, which severely impacts the quality of life of patients^5^.

Salmeterol (Sal) is a common clinical drug in COPD therapy, acting as β2-receptor agonists, which could inhibit early and late antigen-induced airway hyperreactivity ^6–8^. Usually, Sal is combined with glucocorticoid, such as fluticasone, in the prescription for COPD patients. Sal can not only dilate bronchi, but also reduce airway inflammatory response by reducing some inflammatory factors such as interleukin-4 (IL-4)^9^. However, long-term use of this combination could cause adverse reactions and aggravation of the disease, besides its good therapeutic effect in the early stage. Yip et al. also evaluated the effect of changing fluticasone/Sal to mometasone/formoterol treatment in patients with COPD and identified that patients experienced COPD exacerbation after this conversion^10–12^. So, to explore a novel adjuvant drug of Sal is needed.

Magnesium isoglycyrrhizinate (MgIG) has been applied to the clinical treatment of chronic inflammatory disease, such as hepatitis and pneumonia^13,14^, attributed to its glucocorticoid-like chemical structure. In particular, it has been recently recommended as supportive care for mild and common patients of coronavirus disease 2019 (COVID-19)^15^. And, we found that MgIG could alleviate the inflammatory response in COPD rats through reducing the release of the inflammatory cytokines in a COPD rat model^16^.

Besides alternations in alveolar maintenance and abnormal cellular repair, inflammation is a driver of development of COPD. During inflammation, cytokines play an important role. Many cytokines, such as tumor necrosis factor alpha (TNF-α), interleukin-6 (IL-6) and interleukin-1 beta (IL-1β), could activate the Janus tyrosine kinase/signal transducer and activator of transcription (JAK/STAT) signaling pathway, contributing the overexpression of numerous pro-inflammatory factors in the lung^17–19^. So, the JAK/STAT pathway is involved in COPD pathogenesis, and its inhibitors might be a potential treatment for COPD. It follows that MgIG might be a feasible alternative adjuvant drug for glucocorticoids, such as fluticasone propionate, in the treatment of COPD.

In this study, the therapeutic effects of Sal and MgIG on COPD rats exposed to inhaled respiratory lipopolysaccharide (LPS) and cigarette smoke were evaluated, and the underlying mechanisms of JAK/STAT pathway inhibition were also explored.

## Methods

### Preparation of MgIG and other drugs

MgIG injection was purchased from Chia Tai Tianqing Pharmaceutical Group Co., Ltd. (Lianyungang, China). The main chemical composition of this product is MgIG, with the molecular formula of C_42_H_60_MgO_16_·4H_2_O. Sal (CAS:89365-50-4) was purchased from LGM Pharma with purity of 99% and molecular formula of C_25_H_37_NO_4_. Salmeterol Xinafoate and Fluticasone Propionate Powder for Inhalation (50 μg:250 μg) was purchased from Glaxo Operations UK Limited. All drugs were stored at 4 °C prior to use.

### Rat model of COPD and MgIG treatment

The male Wistar rats (body weight, 180 ± 20 g; age, 6–8 weeks) were supplied by the Charles River Laboratory Animal Technology Co., Ltd. (Pinghu, China) (Certificate No. SCXK (Zhe) 2019e001). Experimental procedures involving the use of animals complied with the Guidelines for Animal Experimentation of Jiangsu Vocational College of Medicine. The protocol was approved by the Animal Ethics Committee of Jiangsu Vocational College of Medicine. All animals were raised under standard environmental conditions and provided with a standard laboratory diet and tap water ad libitum.

The rat COPD model was established by smoking and endotracheal atomization (ETA) of endotoxin lipopolysaccharide (LPS) according to previous reports^20^. Rats were randomly divided into groups (10 rats in each group): control group (CON), COPD model group (MDL), Salmeterol xinafoate group (Sal), MgIG group (MgIG), Salmeterol xinafoate and MgIG group (Sal/MgIG), Salmeterol xinafoate and Fluticasone propionate group (Sal/Flu). Except for CON group, rats in other five groups were anesthetized with isoflurane gas on days 1 and 15, followed by a sensitization with 100 μL of 1 mg/mL LPS by an endotracheal-atomization using liquid aerosol devices (MicroSprayer^®^ Aerosolizer, Model IA-1B, Penn-Century, Inc. Wyndmoor, USA), respectively. These rats were also passively exposed to 5% (v/v) cigarette smoke in several fumigation boxes for 30 min twice with an interval of 6 h daily from days 2 to 14. Cigarette smoke was produced by Hademen cigarettes (each contains 10 mg tar, 1.0 mg nicotine content, and 12 mg carbon monoxide) (Jinan, China). As the COPD model rat were obtained, rats were challenged by cigarette smoke alone every day from days 16 to 45 with the method aforementioned. Additionally, Sal group received 0.40 mg/kg/day (w/bw/days) salmeterol xinafoate dissolved in normal saline, MgIG group received 0.40 mg/kg/day (w/bw/days) MgIG, Sal/Flu group received received 0.40 mg/kg/day (w/bw/days) salmeterol xinafoate and fluticasone propionate powder dissolved in normal saline, and Sal/MgIG group received 0.40 mg/kg/day (w/bw/days) salmeterol xinafoate and 0.40 mg/kg/day (w/bw/days) MgIG by endotracheal-atomization 1 h before cigarette smoke treatment from days 30 to 45, respectively, whereas MDL group received 100 μL of 0.9% normal saline with the same delivery method. Rats in CON group were sensitized and challenged with 100 μL of normal saline in the same way and exposed to the normal air. After 45 days, rats were sacrificed with an intraperitoneal injection of pentobarbital sodium (150 mg/kg; Sigma-Aldrich; Merck KGaA). All methods were conducted in accordance with the ARRIVE guidelines (https://arriveguidelines.org).

### Pulmonary function measurement

The pulmonary function of the rats was evaluated using AniRes2005 lung function analysis system (Beilanbo Technology Co., Ltd, Beijing, China) according to the manufacture’s instructions. Briefly, after a 45-day treatment, rats were anesthetized with 3% pentobarbital sodium (70 mg/kg; Sigma-Aldrich; Merck KGaA) and placed in a fixed supine position in a capacity box, which is attached an intubation tube connecting to the ventilator and signal conditioner, transmitting the data of air flow and change in it to the computer. Whereafter, endotracheal intubation was executed on the rats. The respiratory rate and time ratio of expiration/inspiration were preset at 75/min and 1.5:1, respectively^16^. The forced vital capacity (FVC) and forced expiratory volume in 0.3 s (FEV_0.3_) were detected, and the ratio of the two (FEV_0.3_/FVC) was used as the index to evaluate the lung function of rats.

### Cells classification in bronchoalveolar lavage fluid (BALF)

To evaluate airway inflammation, the inflammatory cells accumulated in bronchoalveolar lavage fluid (BALF) were analyzed^16^. Briefly, tracheas of the anesthetized rats were surgically exposed and intubated. The lungs were laved with 3 sequential 1 mL of Hank's balanced salt solution without calcium and magnesium, and BALF was harvested and centrifuged (400*g*) at 4 °C for 10 min. The total number of cells was counted with a standard haemocytometer after resuspension of the cell pellet in 1 mL of Hank's balanced salt solution. Another aliquot of cell resuspensions were used for preparing cell slides, followed by cell staining with Liu's staining solution (PS0290, Beijing Jin Ming Biotechnology Co., Ltd. China). According to the standard morphological criteria, about 200 cells were counted and classified as macrophages, lymphocytes, neutrophils, and eosinophils.

### Measurement of inflammatory factors

To detect inflammatory factors, the serum of rats from all groups was respectively collected. The levels of inflammatory factors, interleukin (IL)-6, interleukin (IL)-1β and tumor necrosis factor (TNF)-α, were determined at 1:40 dilution with ELISA kits (Beyotime, Shanghai, China) following the manufacturer's protocol.

### Histopathological analysis

To evaluate the histopathological changes of lung tissues, the left upper lobe tissues were removed from the anesthetized rats to prepare paraformaldehyde-fixed, paraffin-embedded tissue samples with the regular method. The sample was sliced into pieces with 4 μm-thickness and stained with haematoxylin and eosin (H&E) following standard protocols. The histopathological changes were observed under a light microscope. We randomly selected three visual fields (200×) from each H&E stained section, and then divided the number of alveoli in each visual field by the area of the visual field to obtain MAN (mean alveolar number). Finally, the thickness of bronchial wall was measured directly by 10× microscope. Cell infiltration was scored as follows: 0, no cells; 1, a few cells; 2, a ring of cells and 1 cell deep; 3, a ring of cells and 2–4 cells deep; and 4, a ring of cells and > 4 cells deep. The scores of ten rats were averaged. The histopathological appearance was evaluated independently by three pathologists in a blinded manner.

### Immunohistochemistry

To investigate secretion of mucus in lung of rat in each group, immunohistochemistry assay was executed with mucin MUC5AC antibody ^21^. Briefly, the paraffin-embedded slides were deparaffinized using 100% xylene and rehydrated (100, 95, 90, 85, 80% ethanol). Subsequently, the deparaffinized slides were rehydrated and soaked in sodium citrate buffer to expose antigen, followed by treatment with 3% hydrogen peroxide (10 min) to block endogenous peroxidase. After be washed with PBS, slides were incubated with MUC5AC antibody (Abcam, Cambridge, UK) in 1:500 dilution of original antibody dilution buffer (Beyotime, Shanghai, China) for 12 h at 4 ℃. Secondary antibodies were detected using the SignalStain DAB substrate Kit (cell signaling technology, Beverly, Massachusetts, USA). The slides were counterstained with hematoxylin (Ligen, Beijing, China) and mounted. Each group was examined using the DM6B positive fluorescence microscope (Leica, Frankfurt, Germany). Semi-quantitative image analysis was used to measure the Average Optical Density (AOD) for evaluating MUC5AC expression with Image Pro plus software version 6.0 (Media Cybernetics Corporation, USA).

### Western blotting

The Western blot analysis was performed as described previously^19^. Briefly, the lung tissue was homogenized using lysis buffer with 1 nM phenylmethanesulfonyl fluoride (PMSF) (Beyotime, Shanghai, China), followed by a centrifugation (8000*g*, 10 min) to collect supernatant. The concentration of proteins in the supernatant was determined using a bicinchoninic acid (BCA) protein assay kit (Beyotime, Shanghai, China). For separate proteins, 300 μg proteins from each sample were loaded on a gel for 10% SDS-PAGE. Then, the proteins were transferred to polyvinylidene difluoride (PVDF) membranes and blocked with a 5% bovine serum albumin (BSA) solution for 1 h, followed by an overnight incubation with primary antibodies anti-JAK2 (EPR23073-1, 1:1000, Abcam, Cambridge, United Kingdom), anti-phospho-JAK2 (EPR23028, 1:1000, Abcam, Cambridge, United Kingdom), anti-STAT3 (ab138483, 1:1000, Abcam, Cambridge, United Kingdom) and anti-phospho-STAT3 (ab137521, 1:1000, Abcam, Cambridge, United Kingdom) at 4 °C. Then, membranes were incubated continually with secondary antibodies (1:10,000 dilution in TBST containing 5% skimmed milk), and the bands were detected using a Western blot detection kit (Beyotime, Shanghai, China). The blot was scanned and analyzed using an open source ImageJ software (downloaded for free from https://imagej.nih.gov/ij/) to determine the band densitometry.

### Statistical analysis

All data are presented as the mean ± SD, and analyses were performed with Graphpad Prism software version 8.0 for Win (GraphPad Software, San Diego, CA, USA). Comparisons between experimental groups were conducted using Tukey's after One-way ANOVA for group to group comparison, Values of p < 0.05 were considered significant.

### Ethics approval and consent to participate

Experimental animals were handled under a protocol approved by the Center for Laboratory Animal Care, Jiangsu Experimental Animal Ethics Committee, and Jiangsu Provincial Health Commission of China (Z2019053).

## Results

### The therapeutic effects of Sal and/or MgIG on rats with COPD

**Figure 1:**
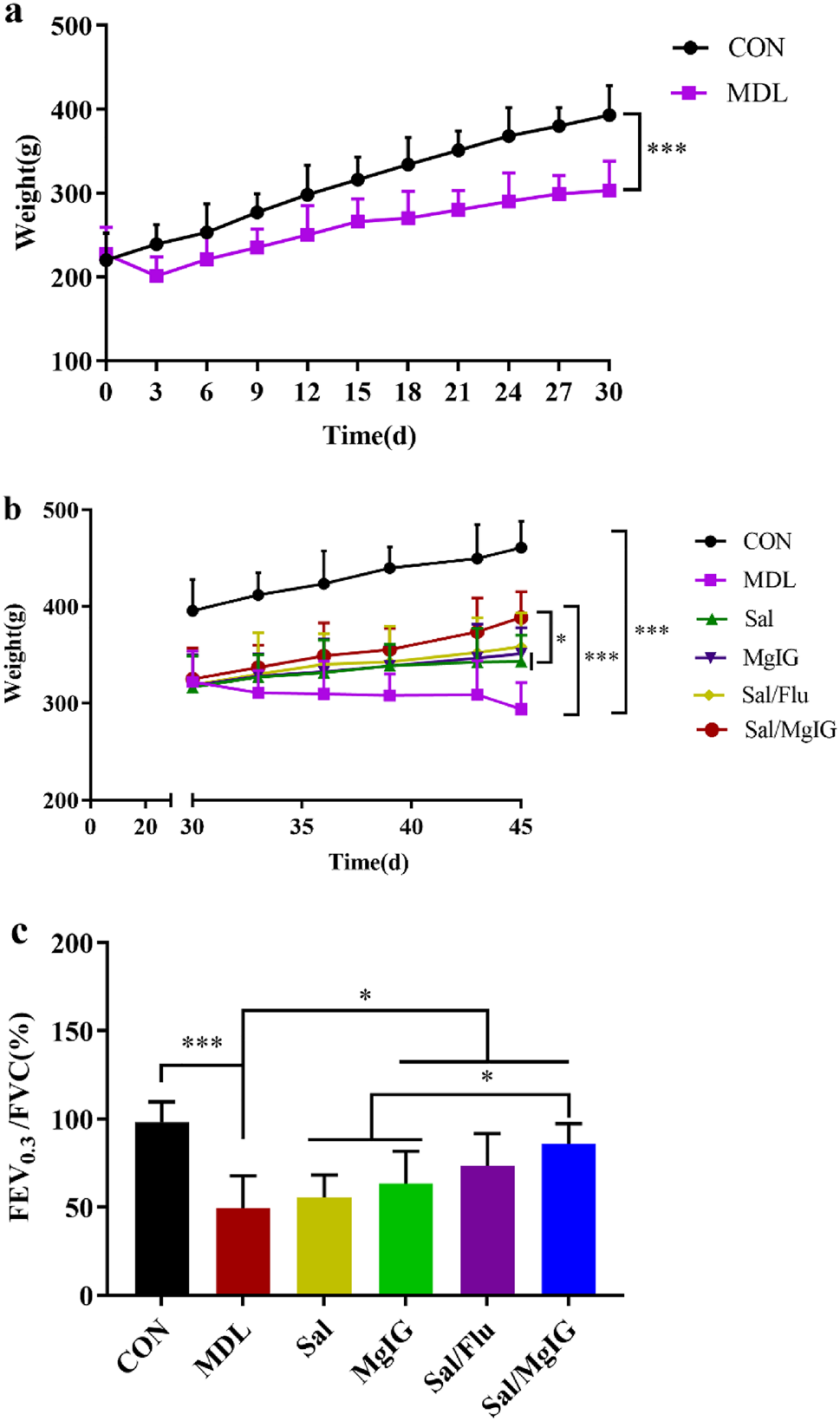
Effects of Sal or/and MgIG on body weight and pulmonary function in COPD rats. (**a**) Changes of body weight of the rats during 30-day COPD model construction. (**b**) Changes of body weight of the rats in different groups during treatment. (**c**) Comparison of FEV_0.3_/FVC of rats in different groups after treatment for pulmonary function evaluation. All data were presented as mean ± standard deviation (n = 10 in each group). Statistical significance of differences was determined by one-way ANOVA. ***p < 0.001, **p < 0.01 and *p < 0.05 was considered to indicate a statistically significant difference. *FEV*_*0.3*_ forced expiratory volume in 0.3 s, *FVC* forced vital capacity.

The body weight of rats in CON group normally increased, while that in MDL group decreased at 3 days of COPD construction, which was significantly recovered after a treatment with salmeterol xinafoate, MgIG, salmeterol xinafoate/MgIG and salmeterol xinafoate/fluticasone propionate drug combination (p < 0.05 or p < 0.01, Fig. 1a,b). In particular, the weight recovery of rats in Sal/MgIG group was better than that in Sal + Flu positive drug combination group. Furthermore, the decrease trend of FEV_0.3_/FVC in MDL group was also reversed after a treatment with salmeterol xinafoate, MgIG, salmeterol xinafoate/MgIG and salmeterol xinafoate/fluticasone propionatedrug combination (p < 0.05 or p < 0.01, Fig. 1c). And, the salmeterol xinafoate/MgIG combination could improve pulmonary function of COPD rats better.

### Levels of inflammatory factors in a rat model of COPD

**Figure 2:**
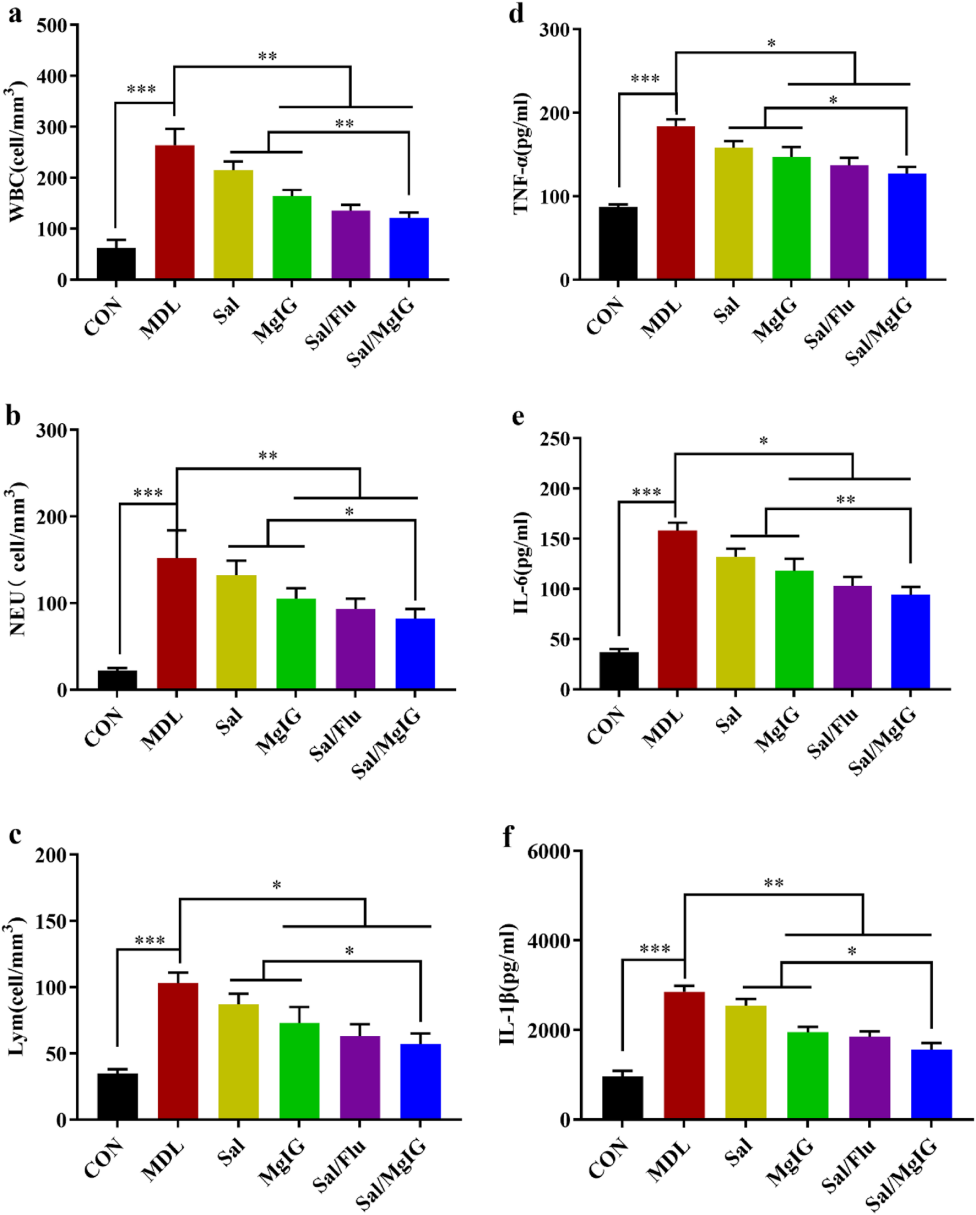
Effects of Sal and/or MgIG on the inflammatory cells in BALF and the levels of inflammatory cytokines in the serum of COPD rats. (**a**) Number of white blood cells in the BALF of rats after 15-day different treatment. (**b**) Number of neutrophils in BALF of rats after 15-day different treatment. (**c**) Number of lymphocytes in BALF of rats after 15-day different treatment. The levels of TNF-α (**d**), IL-6 (**e**) and IL-1β (**f**) in the serum of rats after 15-day different treatment were shown as above. All data were presented as mean ± standard deviation (n = 10 in each group). Statistical significance of differences were determined by one-way ANOVA. ***p < 0.001, **p < 0.01 and *p < 0.05 was considered to indicate a statistically significant difference.

COPD is commonly associated with increased production of inflammatory factors. The levels of inflammatory response cells, such as leukocytes, neutrophils and lymphocytes, in BALF were determined, and results showed that these three types of cells significantly increased during COPD model construction and decreased after a treatment with salmeterol xinafoate, MgIG or salmeterol xinafoate /MgIG or salmeterol xinafoate/fluticasone propionate combination (Fig. 2a–c). Meanwhile, the pro-inflammatory factors (IL-6, IL-1β and TNF-α) in the serum were evaluated, and it showed that the increase trend of these cytokines in MDL group was significantly reversed after a treatment with salmeterol xinafoate, MgIG or salmeterol xinafoate/MgIG or salmeterol xinafoate/fluticasone propionate combination (p < 0.05) (Fig. 2d–f). Among these treatments, salmeterol xinafoate/MgIG combination exhibited a better anti-inflammation effect (p < 0.05).

### Pathological analysis of the effect of salmeterol xinafoate/MgIG treatment in COPD rats

**Figure 3:**
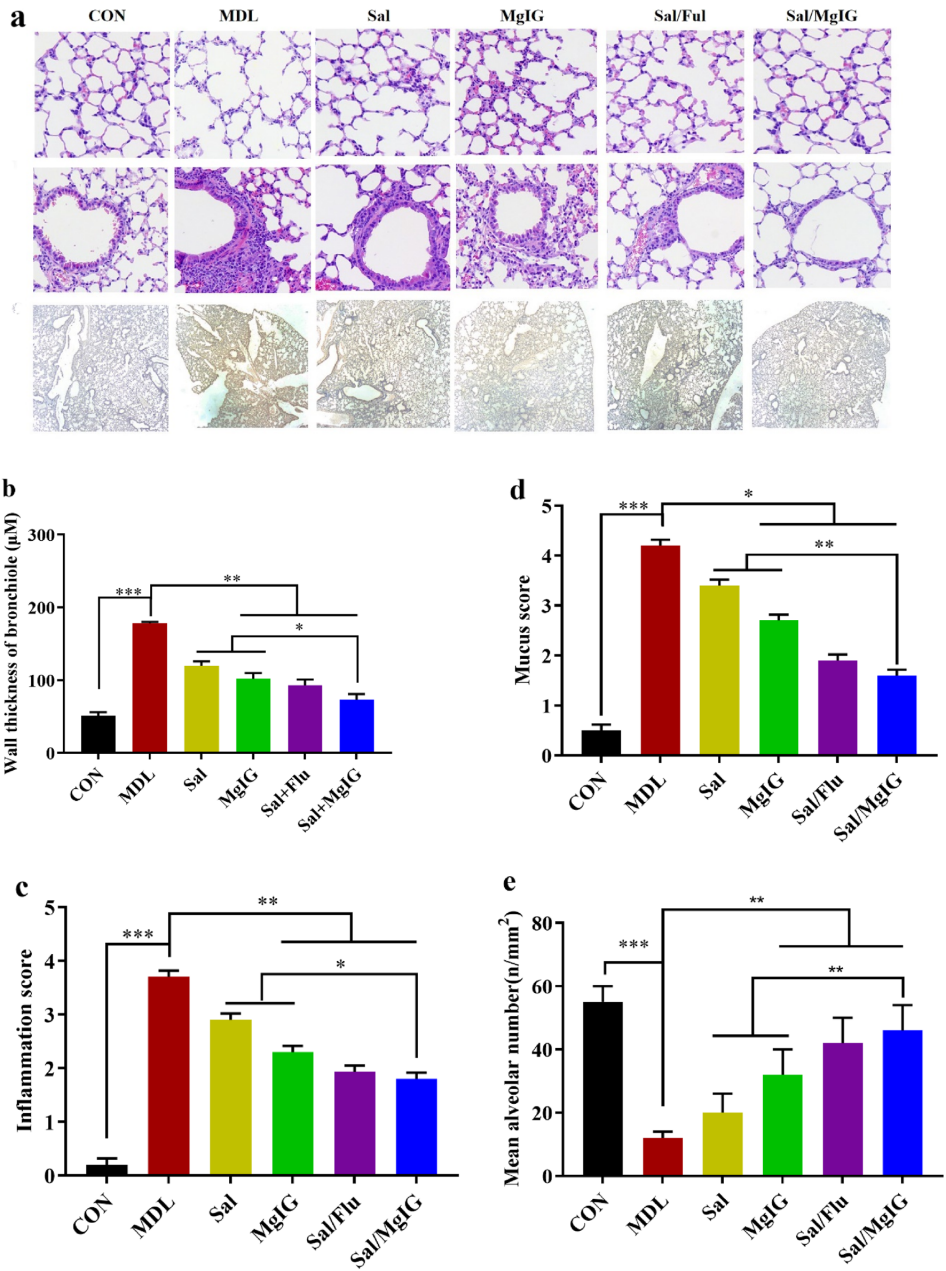
Effect of MgIG and/or Sal on histopathology in rats with COPD. (**a**) The images show the alveoli (H&E ×200) and airway (H&E ×200 or H&E ×100). (**b–e**), bronchial wall thickness, inflammation score, the mean chord length, mucus score and mean alveolar volume were measured to compare the effect of MgIG and/or Sal in rats with COPD. All data were presented as mean ± standard deviation. Statistical significance of differences were determined by one-way ANOVA. ***p < 0.001, **p < 0.01 and *p < 0.05 was considered to indicate a statistically significant difference.

To investigate the pathological changes of airway structure in COPD rats before and after drug intervention, the sections of lungs of rats from each group were prepared and observed after H&E stain and immunohistochemistry (IHC). Compared with MDL group, bronchial wall thickening and mucous plug formation (MUC5AC) was inhibited in all drugs treated rats (Fig. 3a). Salmeterol xinafoate/MgIG treatment also exhibited a marked improvement in pathological changes, including inflammatory cell infiltration reduction, bronchial wall thinning, mean alveoli number decrease, and alveolar dilatation and rupture alleviation (p < 0.05 or p < 0.01, Fig. 3b–e).

### The expression of JAK2/STAT3 in COPD rats

**Figure 4:**
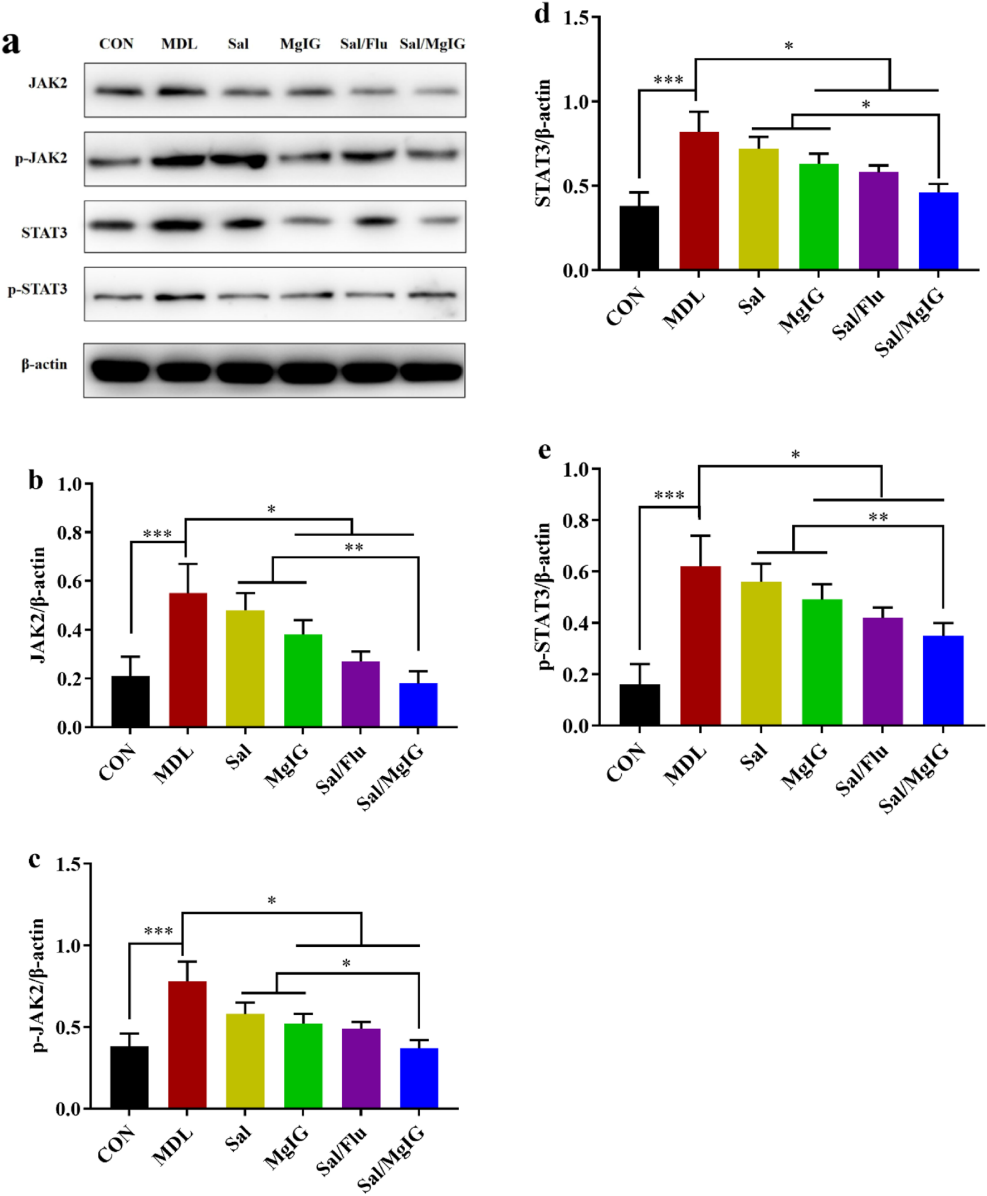
Effect of MgIG and/or Sal on the expression of JAK2, p-JAK2, STAT3 and p-STAT3 in COPD rats. After the rats were sacrificed, the upper lobe of the right lung was removed for western blotting. (**a**) The protein levels of JAK2, p-JAK2, STAT3 and p-STAT3 were detected by western blotting, and the ratio of JAK2/β-actin. (**b–e**), JAK2/β-actin, p-JAK2/β-actin, STAT3/β-actin and p-STAT3/β-actin were shown. All data were presented as mean ± standard deviation (n = 10 in each group). Statistical significance of differences were determined by one-way ANOVA. ***p < 0.001, **p < 0.01 and *p < 0.05 was considered to indicate a statistically significant difference.

Cigarette smoke leads to the activation of JAK2/STAT3 signal path, which may play an important role in the chronic inflammation of COPD. To further examine the mechanism of Sal and/or MgIG treatment on COPD rats model, JAK2/STAT3 level in lung tissues of rats in each group was detected. As demonstrated in Fig. 4a, the results of western blotting analysis showed that the expression levels of JAK2, p-JAK2, STAT3 and p-STAT3 in lung tissue was dramatically decreased after treatment in all drug intervention groups compared to the MDL group. Meanwhile, salmeterol xinafoate/MgIG co-treatment exhibited the most significant effect (p < 0.05 or p < 0.01, Fig. 4b–e).

## Discussion

Exacerbation of COPD is the main cause for its high mortality, approximately 3.23 million deaths in 2019^22^. Many studies suggest that medication with the inhaled corticosteroid fluticasone propionate plus a long-acting beta-agonist salmeterol could reduce the rate of exacerbations and mortality of COPD patients^23^. Ferguson et al., (2003) evaluated the risk of cardiovascular toxicity of salmeterol in COPD patients and found treatment with 50 μg twice daily did not increase the risk of cardiovascular adverse events^24^. Even at dose of 10 mg/kg/day, salmeterol also did not cause any signs of immunotoxicity in rats^25^. However, patients treated with fluticasone propionate reported significantly worsen side effect compared with those treated with ciclesonide, such as perspiration, eye dryness, and tiredness^26^. In this study, rats were treated with salmeterol xinafoate at level of 400 μg/kg/day, which was less than the equivalent dose in human, based on dose conversion between animals and human. Thus, we thought that the treated dose is safe.

MgIG has a variety of pharmacological activities, including anti-inflammatory, antioxidant, antiviral and immune regulation^27,28^. It has a protective effect on the injury of important organs (such as liver and lung), and was recommended as supportive care for mild and common patients of atypical pneumonia^15^. In this study, the rat COPD model was successfully established in terms of increased levels of IL-6 and TNF-α in serum and decline of lung function (FEV_0.3_/FVC ratio), which were used as biomarkers and the main criterion for COPD patients^29^ and well evidenced by the followed hispathological analysis (Fig. 3a). Treatment with salmeterol or MgIG or salmeterol xinafoate/MgIG combination or salmeterol xinafoate/fluticasone propionate combination recovered the body weight loss caused by COPD, and salmeterol xinafoate/MgIG exhibited the best recovery effect, which suggested it could improve the physiological function of COPD rats better. Meanwhile, it also showed that the co-treatment of salmeterol xinafoate/MgIG could significantly improve the pulmonary function of COPD rats contributing to their increased vital capacity. This might attribute to its effect on infiltration of inflammatory cells alleviation and mucus secretion reduction (Figs. 2 and 3). So, the salmeterol xinafoate/MgIG combination could play a better synergistic effect than the positive combination of salmeterol xinafoate/fluticasone propionate.

Pro-inflammatory factors play an important regulatory role in the progression of COPD. The further investigation revealed that the pro-inflammatory factors TNF-α, IL-6, and IL-1β levels decreased significantly in the serum of rats from group treated with drugs, following the order: salmeterol xinafoate/MgIG > salmeterol xinafoate/fluticasone propionate > salmeterol xinafoate > MgIG. JAK-STAT signal pathway plays an important role in regulating inflammatory response and immune response in COPD related studies, especially expression and tyrosine phosphorylation of STAT3^17,18,30,31^. And inflammatory factor TNF-α, IL-6, and IL-1β could activate JAK2 and STAT3, which could further promote other pro-inflammatory factors expression to exacerbate inflammation^32,33^. Our analysis showed that JAK2 and STAT3 expression and phosphorylation decreased along with the reduction of inflammatory factors TNF-α, IL-6, and IL-1, thereby inhibiting the overexpression of numerous other pro-inflammatory factors in the lung, which needs more evidence to verify in further studies.

## Conclusion

It indicated that salmeterol xinafoate/MgIG drug combination could play a better synergistic effect to alleviate pulmonary inflammation and improve lung function of COPD rats by JAK/STAT pathway, and MgIG might be a potential clinical alternative adjuvant drug for fluticasone propionate in routine clinical drug combination of salmeterol xinafoate/fluticasone propionate for COPD treatment (Supplementary information).

## Supplementary Information

Supplementary Information.

## Data Availability

The datasets used and/or analyzed during the current study are available from the corresponding author on reasonable request.
