# Supplementary material for: Efficacy of salmeterol and magnesium isoglycyrrhizinate combination treatment in rats with chronic obstructive pulmonary disease
Source: Sci Rep. 2022 Jul 19;12:12334. doi: 10.1038/s41598-022-16775-2 (PMC9296643; doi:10.1038/s41598-022-16775-2)
Supplement: Supplementary file 1 — Supplementary Information. [file 41598_2022_16775_MOESM1_ESM.pdf]

Efficacy of salmeterol and magnesium isoglycyrrhizinate combination treatment in rats with chronic obstructive pulmonary disease

Ye Yang, Lei Huang, Chongchong Tian, Bingjun Qian*

Department of Pharmacology and Medicinal Chemistry, Jiangsu Vocational College of Medicine, Yancheng, Jiangsu 224005, P.R.China

Figure 4 JAK2,p-JAK2,STAT3,p-STAT3

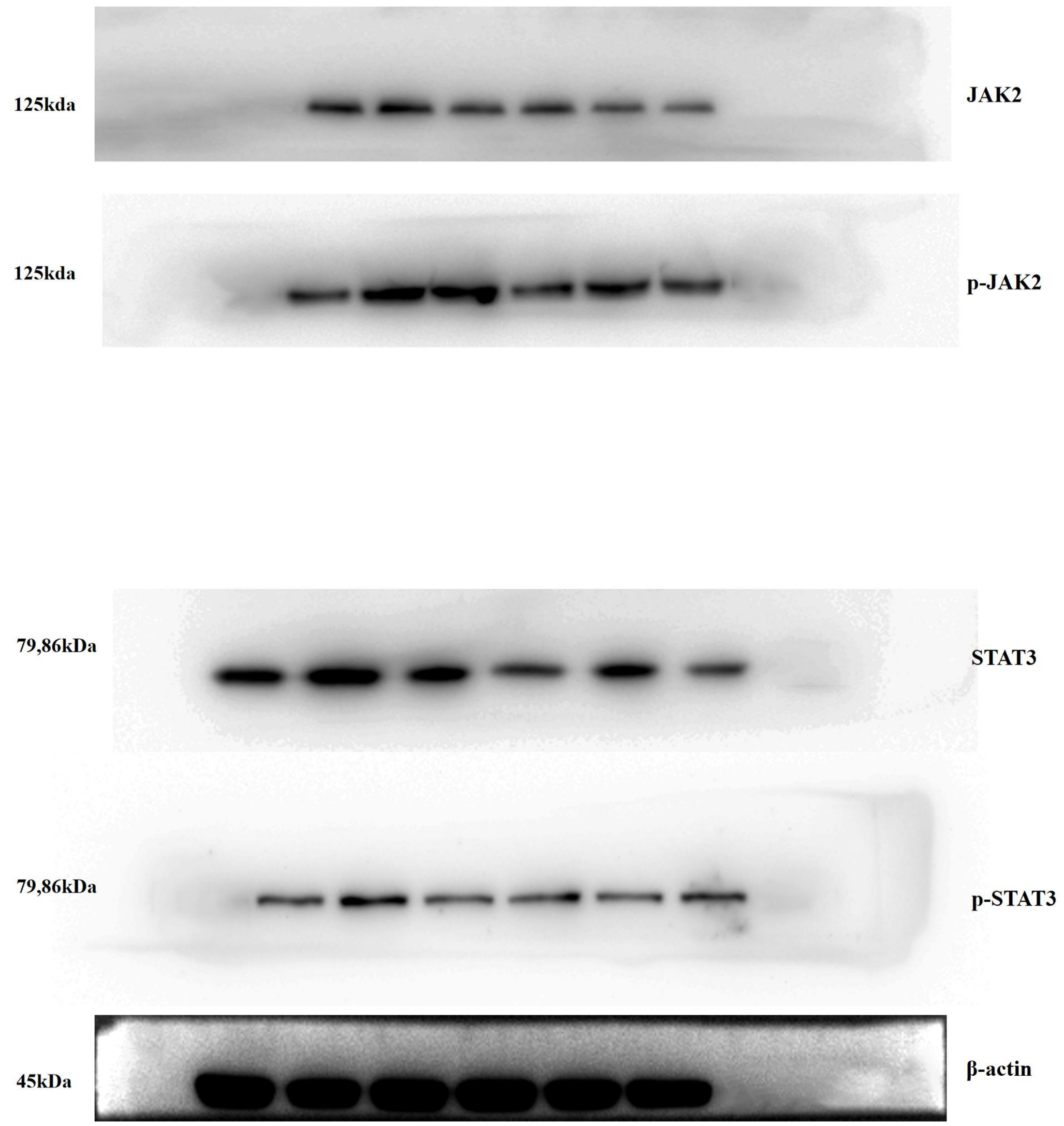

There are full-length gels and blots of JAK2 (first row), its p-JAK2 (second row), STAT3 (third row), and its p-STAT3 (fourth row) in Figure 4. In the four gels, from left to right, the first blots represent the expression level in the lung of control rats, the second blots represents the expression level in the lung of model rats (MDL), the third blots represents the expression level in the lung of Sal rats, the fourth blots represents the expression level in the lung of MgIG rats, the Fifth blots represents the expression level in the lung of Sal+Flu rats, and the last blots represents the expression level in the lung of Sal+MgIG rats.
